# Socioeconomic inequalities in health care utilisation in Norway: the population-based HUNT3 survey

**DOI:** 10.1186/1475-9276-11-48

**Published:** 2012-08-22

**Authors:** Eirik Vikum, Steinar Krokstad, Steinar Westin

**Affiliations:** 1 Department of Public Health and General Practice, Faculty of Medicine, Norwegian University of Science and Technology (NTNU), MTFS, 7489, Trondheim, Norway; 2 HUNT Research Centre, Department of Public Health and General Practice, Faculty of Medicine, Norwegian University of Science and Technology (NTNU), Trondheim, Norway; 3 Levanger Hospital, Nord-Trøndelag Health Authority, Trondheim, Norway

**Keywords:** Social inequalities, Health care, Health services research, Utilisation, Public health, Norway

## Abstract

**Background:**

In this study we investigated the distribution of self-reported health care utilisation by education and household income in a county population in Norway, in a universal public health care system based on ideals of equal access for all according to need, and not according to wealth.

**Methods:**

The study included 24,147 women and 20,608 men aged 20 years and above in the third Nord-Trøndelag Health Survey (HUNT 3) of 2006–2008. Income-related horizontal inequity was estimated through concentration indexes, and inequity by both education and income was estimated as risk ratios through conventional regression.

**Results:**

We found no overall pro-rich or pro-educated socioeconomic gradient in needs-adjusted utilisation of general practitioner or inpatient care. However, we found overall pro-rich and pro-educated inequity in utilisation of both private medical specialists and hospital outpatient care. For these services there were large differences in levels of inequity between younger and older men and women.

**Conclusion:**

In contrast with recent studies from Norway, we found pro-rich and pro-educated social inequalities in utilisation of hospital outpatient services and not only private medical specialists. Utilisation of general practitioner and inpatient services, which have low access threshold or are free of charge, we found to be equitable.

## Introduction

While the major sources of social inequalities in health are considered to arise from social and economic determinants outside the health services [1], there is an increasing interest in the role of the health care system. In the Scandinavian countries and other countries with universal health care systems, it is generally assumed that socioeconomic gradients in access to health care are very low or non-existent, and only recently has this been subject to critical review [2]. Even without a precise quantification of the contribution to health equity by the health services however, there is an obvious need to study the social patterning of utilisation of health care.

The availability of good medical care tends to vary inversely with the need for it in the population served, wrote Julian Tudor Hart in 1971 [3]. Recent international studies have found that many Western countries have socioeconomic equity in utilisation of primary care, whereas more pro-rich inequity arises in the use of services from medical specialists [2,4,5]. The same pattern has been described in the universal health care system of Norway, most recently by Grasdal and Monstad, who found that the national implementation of a list-based system for general practitioners in 2001 evened out some of the previously found inequalities in access to private specialist services [6]. Grasdal and Monstad did not find inequity in hospital outpatient services, however, in agreement with a study by Iversen and Kopperud from 2005 [7]. Both of these studies used Norwegian Survey of Living Conditions-data that is nation-wide, but based on small samples compared to the population-based third Nord-Trøndelag Health Survey (HUNT3) of 2006–08. The larger sample size in HUNT3 facilitates a more detailed analysis of inequity in subgroups of age and gender.

In recent years an increasing number of studies have employed a concentration index methodology to calculate income-based horizontal inequity (HI index) in health care utilisation [4,5,8-10]. In the present study of socioeconomic gradients in health care utilisation we estimated both risk ratios and horizontal inequity indexes in order to ease interpretation and to facilitate comparison with other studies.

### The Norwegian health care system

The Norwegian health care system is currently, as in 2006–08, characterized by universal coverage and public provision of services. In 2001 a list-based system for patients in general practice was introduced, assigning nearly all citizens to specific general practitioners. Most GPs are self-employed on contracts with municipalities, and are thus considered part of the public health care system. Similarly most medical specialist practices outside hospitals are private, but operate on contracts with the public Regional Health Authorities. Fully private general practitioners, specialist services and hospitals exist, but to a very minor degree, and mainly in urban areas. In this study, ‘private medical specialist’ refers to all medical specialists outside hospitals, the vast majority of whom were self-employed but publicly contracted when the survey was conducted.

General practitioners are gatekeepers to all specialist care and elective hospital treatment that is reimbursed by the National Insurance Scheme. In general, copayments for GP services, publicly contracted specialists and outpatient hospital treatment are fixed, the level in the survey period approximately NOK 170 for general practitioner consultations and NOK 270 for specialist consultations. The cap on yearly co-payment for patients was at approximately NOK 1600 when the data were gathered, anything beyond that amount covered by the National Insurance Scheme. Public hospital inpatient care is free.

### Aims

In this study the aim was to investigate the distribution of self-reported health care utilisation by education and household income in a county population in Norway.

## Material and methods

### Data sources

The Nord-Trøndelag Health Study (HUNT) is a total county population based health survey, and maintains a unique database of medical histories collected during three cross-sectional surveys: HUNT1 (1984–1986), HUNT2 (1995–1997) and HUNT3 (2006–2008) [11].

Nord-Trøndelag is one of 19 counties in Norway, situated in the middle of the country. Its geography and demography is considered to be representative of Norway as a whole, with a stable and homogenous population of approximately 127 000 inhabitants [12]. Socioeconomic inequalities in health and mortality in Nord-Trøndelag have been demonstrated in several studies [13,14]. The county lacks large cities, however, and social inequalities may thus be smaller than in the country as a whole due to the larger inequalities usually found in cities. The level of average income is somewhat lower than the average of Norway [15].

All persons aged 20 years and above in the county of Nord-Trøndelag were invited to participate in the HUNT 3 Survey (2006–08). Out of 94,191 persons invited, 54% (50,797) responded to the questionnaire used in this study. Response rates were 37% among men and women 20–39 years old, 60% among men and women 40–59 years and 63% among those over 60 years. Health and utilisation data were taken from HUNT3 (2006–2008), while household income and level of education was appended from national register data from Statistics Norway (SSB) using the unique personal identity number given all Norwegian citizens.

We restricted analyses to men and women 20 years and older. 20,608 males and 24,147 females were included in the study. 6,042 respondents were excluded due to incomplete data for variables from either HUNT3 (n = 5,458) or Statistics Norway (n = 584).

### Variables

Four dichotomous indicators of health care utilisation were employed. Respondents were asked if they in the past 12 months had or had not visited a general practitioner, a medical specialist outside a hospital, attended a non-psychiatric outpatient consultation at a hospital, or received inpatient care. Some of the respondents who had missing responses on the questions regarding medical specialist and outpatient consultation, had responded positively to having used at least one of the other forms of health care in the proximity of these questions in the questionnaire, and negatively to none. In these cases (n = 7,276 and n = 6,356, respectively), the missing values were judged to be due to a misunderstanding resulting from the survey design, and non-response was treated as nonattendance.

For needs adjusting in the analyses of health care utilisation, the following variables were used: Self-reported health was measured by four response alternatives: “very good”, “good”, “poor” and “very poor”, as well as the dichotomous “Do you suffer from long-term (at least one year long) illness or injury of a physical or psychological nature that impairs your functioning in your daily life?” In addition, the respondents were asked to indicate past or present suffering from 18 different long-term conditions, including cancer, diabetes and heart conditions. Persons with missing response to one or some of the morbidity questions were treated as not having that specific disease or impairment, if they had responded to at least one indicator.

Educational level, obtained from Statistics Norway and following the Norwegian Standard Classification of Education (NUS), was coded into three levels of highest educational level attained: primary (primary and secondary school), secondary (high school or equivalent) and tertiary (college and/or university), primary used as reference category. Municipality of residence for each respondent was included in the analyses as a measure to control for regional differences in access to health services. The variable subdivided respondents into three categories: municipalities with less than 10,000 inhabitants (*n = 19*), large (more than 10,000 inhabitants) municipalities without hospital (*n = 3*), and large municipalities with hospital (*n = 2*).

Disposable income per equivalent adult was calculated using household income after tax from 2007 based on tax registry data from Statistics Norway. Where available, spouses and cohabiting persons over 18 years were given a weight of 0.5, and children up to 18 years a weight of 0.3. The lowest income quartile was used as the reference. Separate income quartiles and income rankings were calculated within age groups and gender where applicable.

### Statistical analysis

Horizontal inequity indexes (HI index) were used to describe income-related inequity, and risk ratios (RR) were used for inequity relating to both level of education and income. Risk ratios were estimated using a modified Poisson regression model with robust error variances [16]. Risk ratios were estimated rather than odds ratios, which would be more difficult to compare across the utilisation indicators due to the large variation in average attendance (Table 1). Separate analyses were performed in three age groups to explore intergenerational variation in inequity, while 10-year intervals were used for age adjustment in the statistical analyses.

**Table 1:** Overview of health care attendance (%) last 12 months. Men and women 20 years and older

	***%***		**GP**		**Inpatient care**		**Outpatient care**		**Med. spec.**	
	**Men**	**Women**	**Men**	**Women**	**Men**	**Women**	**Men**	**Women**	**Men**	**Women**
*Education*										
Primary	20	23	81	88	14	14	25	25	13	15
Secondary	58	48	76	86	11	12	24	27	13	16
Tertiary	22	29	68	80	8	11	25	29	14	15
*N*	*20,608*	*24,147*								
*Household income*										
1st quartile	25	25	80	87	14	14	24	25	14	15
2nd quartile	25	25	78	86	11	13	26	28	14	15
3rd quartile	25	25	74	85	10	12	26	29	13	15
4th quartile	25	25	70	81	9	11	22	27	12	15
*N*	*20,608*	*24,147*								
*Age groups*										
20-39 yrs	20	24	64	82	7	15	17	22	11	12
40-59 yrs	44	43	71	82	9	9	24	28	12	14
≥ 60 yrs	35	33	87	90	15	14	30	29	15	18
*N*	*20,608*	*24,147*								
*Health*										
Very poor	1	1	97	97	38	34	56	52	29	32
Poor	22	27	92	95	19	18	39	39	20	22
Good	61	56	74	84	9	10	22	24	12	13
Very good	16	16	56	70	5	7	13	17	8	8
N	*20,608*	*24,147*								

Horizontal inequity indexes were estimated through convenient regressions following O’Donnell et al. [17] See Van Doorslaer et al. 2006 [9] or Masseria and Giannoni 2010 [8] for additional introductions to the method. The indexes were not sensitive to whether linear or non-linear regressions were used in needs adjustment, and a probit regression was utilised for the indexes presented here.

The horizontal inequity index is a version of the concentration index, which expresses the income-rank distribution of a given variable in a single number. A horizontal inequity index is a concentration index of specifically needs-adjusted health care utilisation, as opposed to “crude” utilisation, which is unadjusted for health care need. In this context, the term ‘horizontal’ refers to inequalities in utilisation that cannot be explained by differences in need, but that can be explained by differences in levels of socioeconomic status. In contrast, ‘vertical’ inequity would refer to inequalities in health care utilisation that are due to inequalities in need for health care, and could be considered fair or just.

In practice need is defined as expected utilisation given certain available indicators, in this study age, gender, self-reported health status and a set of indicators of morbidity. In this context adjusting for need means that we predict the probability of health care utilisation for each person based on these need characteristics, while holding other non-need variables such as education and income constant at their means. When need-predicted utilisation is subtracted from crude utilisation, we gain a measure of the residual inequality that cannot be explained by the observed differences in need (the HI index).

When the concentration index (CI) of crude utilisation is positive, unadjusted (“actual”) health care utilisation is distributed pro-rich, and vice versa it is negative when the distribution is pro-poor. When the CI of need-predicted use is negative, need for health care is concentrated among the poorer. Finally a positive HI index indicates that rich persons utilise health care at higher rates than would be predicted from the available need indicators, relative to the poor.

Separate HI indexes were also estimated within three age groups for both men and women. Weights were not used. All analyses were performed using Stata/IC 10.0.

The study was approved by the Regional Committee for Medical Research Ethics and the Norwegian Data Inspectorate Board.

## Results

Table 1 shows an overview of unadjusted health care utilisation by age, education, household income quartiles and self-reported health.

The concentration indexes in Table 2 show that the distribution of unadjusted health care utilisation among men and women aged 20 years and above was pro-poor for general practitioners, private medical specialists and inpatient care, and equal for outpatient care. The concentration indexes of need-predicted health care utilisation show that the need for all health services was concentrated among the poorer, but less so for general practitioners. The horizontal inequity indexes suggest that the probability of at least one visit to a general practitioner and inpatient care utilisation was equitably distributed by income, whereas the utilisation of private medical specialists and outpatient consultations was distributed pro-rich.

**Table 2:** **HI indexes and relative risks**^**a **^**(RR) for probability of health care utilisation last 12 months. Men and women, 20 years and older**

		**GP**		**Inpatient**		**Outpatient**		**Med. spec.**	
		**(n = 44755)**		**(n = 44755)**		**(n = 44755)**		**(n = 44755)**	
		**Coeff.**	**s.e.**	**Coeff.**	**s.e.**	**Coeff.**	**s.e.**	**Coeff.**	**s.e.**
CI (actual)		**−0.02**	0.00	**−0.07**	0.01	0.00	0.00	**−0.01**	0.01
CI (need-predicted)		**−0.02**	0.00	**−0.08**	0.00	**−0.04**	0.00	**−0.06**	0.00
HI index		0.00	0.00	0.01	0.01	**0.04**	0.00	**0.05**	0.01
	Mean	RR	s.e.	RR	s.e.	RR	s.e.	RR	s.e.
Primary edu	*0.21*	1.00	/	1.00	/	1.00	/	1.00	/
Secondary edu	*0.53*	1.01*	0.01	0.96	0.03	1.17**	0.04	1.18**	0.04
Tertiary edu	*0.26*	0.98*	0.01	0.96	0.04	1.43**	0.05	1.36**	0.05
Income q1	*0.25*	1.00	/	1.00	/	1.00	/	1.00	/
Income q2	*0.25*	1.02**	0.01	1.05	0.04	1.16**	0.03	1.03	0.03
Income q3	*0.25*	1.02**	0.01	1.01	0.04	1.19**	0.04	1.10**	0.04
Income q4	*0.25*	0.99	0.01	0.98	0.04	1.13**	0.04	1.09*	0.04
Female	*0.54*	1.00	/	1.00	/	1.00	/	1.00	/
Male	*0.46*	0.89**	0.00	0.88**	0.02	0.94**	0.02	0.91**	0.02
Age 20-39	*0.22*	1.00	/	1.00	/	1.00	/	1.00	/
Age 40-59	*0.43*	0.99	0.01	0.64**	0.02	1.13**	0.03	0.98	0.03
Age ≥ 60	*0.34*	1.06**	0.01	0.69**	0.03	1.03	0.04	1.02	0.04
Muni small	*0.35*	1.00	/	1.00	/	1.00	/	1.00	/
Muni large	*0.44*	1.04**	0.01	0.95	0.03	1.01	0.03	1.14**	0.03
Muni w/hospital	*0.22*	1.02*	0.01	1.01	0.04	1.02	0.03	1.10**	0.03
Very good halth	*0.16*	1.00	/	1.00	/	1.00	/	1.00	/
Good health	*0.58*	1.19**	0.01	1.33**	0.07	1.40**	0.06	1.30**	0.06
Poor health	*0.24*	1.28**	0.01	2.00**	0.11	1.88**	0.08	1.63**	0.08
Very poor health	*0.01*	1.28**	0.02	3.18**	0.25	2.32**	0.17	2.15**	0.17
No func impairment	*0.59*	1.00	/	1.00	/	1.00	/	1.00	/
Func impairment	*0.41*	1.08**	0.01	1.38**	0.05	1.39**	0.05	1.61**	0.05
Cancer^b^	*0.05*	1.06**	0.01	1.58**	0.07	1.67**	0.05	1.31**	0.05
Respiratory disease^b,c^	*0.11*	1.07**	0.01	1.16**	0.04	1.13**	0.04	1.12**	0.04
CVD^b,c^	*0.09*	1.09**	0.01	1.76**	0.06	1.16**	0.04	1.10**	0.04
Skin disease^b,c^	*0.16*	1.03**	0.01	0.99	0.03	1.09**	0.03	1.23**	0.03
Musculoskeletal^b,c^	*0.21*	1.03**	0.00	1.06	0.03	1.22**	0.03	1.23**	0.03
Diabetes^b^	*0.04*	1.10**	0.01	1.14*	0.06	1.26**	0.06	1.23**	0.06
Renal disease^b^	*0.02*	1.02	0.01	1.44**	0.08	1.22**	0.07	1.05	0.07
Epilepsy^b^	*0.01*	1.01	0.02	1.08	0.11	1.27**	0.11	1.26**	0.11
Stroke^b^	*0.02*	1.06**	0.01	1.55**	0.09	1.13**	0.06	1.02	0.06

** P < 0.01.

* P < 0.05.

Statistically significant concentration indexes and HI indexes in bold.

^a^ Risk ratios and HI indexes were adjusted for all variables in the table.

^b^ The reference for the disease-specific RRs is the absence of that or those diseases.

^c^ Respiratory disease: asthma, COPD, chronic bronchitis, emphysema. Cardiovascular disease: Angina pectoralis, myocardial infarction, heart failure, or other heart disease. Musculoskeletal: rheumatoid arthritis, ankylosing spondylitis, osteoporosis, osteoarthritis, fibromyalgia. Skin: eczema or psoriasis.

The risk ratios presented in Table 2 mirror the pattern in the HI indexes. Pro-rich income and educational gradients are present for private and hospital specialist services among men and women aged 20 years and above, but not for general practitioners and inpatient care. Poor health, functional impairment and morbidity is associated with higher probability of utilisation of all services. The inhabitants of the largest municipalities have higher probabilities of having visited a general practitioner or a private medical specialist.

### Within age groups

Table 3 shows that the probability of at least one visit to a general practitioner is generally equitably distributed by education and income within each age group. The exception is among men aged 20–39, where men with higher education are less likely to see a general practitioner than men with low education.

**Table 3:** **Relative risks**^**a **^**(RR) and HI indexes for health care utilisation last 12 months, by gender and age groups**

		**Men**		**Women**		**Men**		**Women**	
		**RR / HI**	**s.e.**	**RR / HI**	**s.e.**	**RR / HI**	**s.e.**	**RR / HI**	**s.e.**
		**GP**				**Inpatient care**			
Age 20–39 years									
N		*(n = 4161)*		*(n = 5789)*					
HI index		0.01	0.01	0.00	0.00	0.00	0.03	**0.06**	0.02
Education	*Primary*	1.00	/	1.00	/	1.00	/	1.00	/
	*Secondary*	0.92*	0.03	0.98	0.02	0.83	0.12	0.88	0.09
	*Tertiary*	0.79**	0.03	0.97	0.02	0.69**	0.13	0.97	0.10
Age 40–59 years									
N		(n = 9148)		(n = 10286)					
HI index		−0.01	0.00	0.00	0.00	−0.02	0.02	0.01	0.02
Education	*Primary*	1.00	/	1.00	/	1.00	/	1.00	/
	*Secondary*	1.01	0.02	1.02	0.01	0.84*	0.07	0.97	0.07
	*Tertiary*	0.96	0.02	0.99	0.01	0.64**	0.07	1.00	0.09
Age ≥ 60 years									
N		(n = 7299)		(n = 8072)					
HI index		0.00	0.00	0.00	0.00	−0.01	0.02	0.00	0.02
Education	*Primary*	1.00	/	1.00	/	1.00	/	1.00	/
	*Secondary*	1.04**	0.01	1.02*	0.01	1.02	0.06	0.98	0.06
	*Tertiary*	1.00	0.02	1.00	0.01	0.97	0.09	0.93	0.09
		**Outpatient consultation**				**Medical specialist**			
Age 20–39 years									
HI index		−0.03	0.02	**0.03**	0.01	0.02	0.03	0.01	0.02
Education	Primary	1.00	/	1.00	/	1.00	/	1.00	/
	Secondary	0.95	0.09	0.92	0.07	1.21	0.16	1.03	0.11
	Tertiary	0.95	0.11	1.19*	0.10	1.04	0.16	1.17	0.13
Age 40–59 years									
HI index		**0.02**	0.01	**0.02**	0.01	**0.04**	0.02	**0.04**	0.01
Education	Primary	1.00	/	1.00	/	1.00	/	1.00	/
	Secondary	1.08	0.05	1.11*	0.05	0.93	0.07	1.25**	0.08
	Tertiary	1.27**	0.07	1.38**	0.06	1.16	0.10	1.34**	0.10
Age ≥ 60 years									
HI index		**0.08**	0.01	**0.07**	0.01	**0.06**	0.02	**0.08**	0.01
Education	Primary	1.00	/	1.00	/	1.00	/	1.00	/
	Secondary	1.20**	0.05	1.28**	0.05	1.28**	0.09	1.26**	0.07
	Tertiary	1.50**	0.08	1.65**	0.09	1.72**	0.15	1.62**	0.12

* P < 0.05.

** P < 0.01.

Statistically significant HI indexes in bold.

^a^ Risk ratios estimated using same Poisson regression and model as presented in Table 2.

For inpatient care the breakdown into age groups reveals pro-rich inequity among women aged 20–39 years. The risk ratios show that among men aged 20–39 and 40–59 years, low education is associated with a higher probability of having received inpatient care within the past year.

Utilisation of hospital outpatient consultation was pro-rich and pro-educated for all age-sex groups except men aged 20–39 years. Private medical specialist utilisation was pro-rich among men and women aged 40–59 years and over 60 years. The risk ratios reveal a pro-educated gradient among men and women aged 60 and above, and among women aged 40–59 years.

### Missing data

Because the data appended from Statistics Norway were more complete than the HUNT3 data except for the gender variable, we could see that exclusion due to missing variables in HUNT3 was correlated with being male, having low education and belonging in the lowest household income group.

Non-response in the utilisation variables for outpatient consultation and private medical specialist was correlated with lower education, higher age and being male. In sensitivity analyses, the overall estimates of inequity in this study were not sensitive to the inclusion of the cases where non-response was treated as nonattendance. The magnitudes of some subgroup estimates were sensitive to a minor degree, but the direction of the estimates and the general pattern were robust.

## Discussion

Our data show a tendency toward pro-rich income-related inequity in utilisation of private medical specialist services and hospital outpatient services. We found no evidence for overall pro-rich or pro-educated inequity in probability of utilisation of general practitioner and inpatient care. Analyses within age-sex groups revealed that in the older part of the population, the levels of income and education inequity in utilisation of specialist services were higher than the overall levels. In general, inequity in utilisation among young men and women was small or absent. Notable exceptions were a lower probability of general practitioner attendance and inpatient care among higher-educated young men, and pro-rich inequity in "probability inpatient care among women of child-bearing age".

We found significant education and income-related inequity in utilisation of hospital outpatient services both overall and within certain age groups, in contrast with Grasdal and Monstad [6] and Iversen and Kopperud [7] who used the smaller, but nation-wide Norwegian Survey of Living Conditions of 2005 and 2000, respectively. Our overall findings do not conflict strongly with earlier studies for probability of visit to general practitioner, private medical specialists and inpatient care. Income inequity in private specialist care is slightly lower than that of just under 0.06 for Norwegian specialist services (outpatient and private combined) reported in van Doorslaer et al. 2006 [9], and close to the 0.05 estimate of Grasdal and Monstad. The level of income inequity in specialist utilisation confirmed here is moderate compared with other Western countries as reported by Van Doorslaer et al. 2006.

Norwegian general practitioners play a gatekeeper role in access to specialist care, and factors pertaining to both the patient and the GP, or other aspects of the doctor-patient interaction at the primary level, may be involved in creating the inequities we observe. Our study suggests that the level of utilisation of general practitioner services in the Norwegian health care system is generally high, and that utilisation is equitably distributed by income and education. Efforts to reduce socioeconomic inequity in health care utilisation in Norway should therefore focus on identifying mechanisms that either promote, or fail to prevent, inequities that arise in the general practitioner consultation or in the management of referrals to both private and hospital specialist services. A recent Norwegian study has shown that general practitioners allow for more time during consultations with people with high socioeconomic status [18], providing one possible mechanism.

The direction and magnitude of the education-related risk ratios and the income-related horizontal inequity indices in this study generally agree. There are exceptions, however, notably in inpatient care. The results suggest that while education and income in many cases are comparable as socioeconomic indicators in the context of health care utilisation, equity in the one socioeconomic dimension does not rule out inequity in the other. The variation in the type and level of inequity revealed in the subgroup analyses by age and gender further emphasises the disadvantage of one-dimensional and non-stratified analysis of socioeconomic inequalities in health care utilisation.

We found the population size of the municipalities to be positively correlated with probability of utilisation of general practitioner care and private medical specialists. There are several possible explanations. Greater travel distances for inhabitants of rural municipalities to both local general practitioners and to private medical specialists that are typically located in the larger municipalities could represent lower accessibility. Furthermore, rural municipalities often have more difficulty recruiting general practitioners, which could lead to doctor-patient relationships that are less stable due to higher turnover, or less efficient due to recruiting of doctors who do not speak Norwegian well, in turn leading to lower utilisation. Residing in a municipality with one of the two hospitals in Nord-Trøndelag was not correlated with higher probability of outpatient or inpatient care. This could suggest equal distribution of hospital resources between municipalities in the region.

### Strengths and limitations

The strengths of this study include the size of the HUNT3-survey, and the population based data. The health trends of the Nord-Trøndelag region have previously been found to be close to representative of the Norwegian population [19]. With small towns and rural areas with long travel distances, the county is in many ways comparable to the country as a whole. The income and education variables from Statistics Norway are considered accurate.

The lower participation rate in HUNT3 (54%) than in the earlier HUNT surveys is a limitation. A new nonparticipation study for the HUNT3 Survey found that non-responders were more likely to have low education and low income, higher mortality and higher prevalence of cardiovascular disease, diabetes and psychiatric disorders, compared to responders (Langhammer A, personal correspondence, 14^th^ June, 2012). Musculoskeletal pain, urine incontinence and headache were found to be less prevalent among non-responders. Overall, responders and non-responders had comparable unadjusted levels of at least one visit to a general practitioner in the past year, while both male and female non-responders had higher probabilities of inpatient care utilisation. This nonparticipation study also found consistent levels of self-reported utilisation of general practitioner services in different age groups in HUNT3 when comparing with data extracted from general practices in the county.

We consider it most likely that the association between non-response and low education and income has led to underestimation of pro-rich and pro-educated inequity in this study. On the one hand, the higher levels of inpatient care utilisation found among non-responders, who were also found to have lower average education and income than responders, is a potential source of bias that could have led to overestimation of the inequity reported in this study, in a pro-rich and pro-educated direction. However, the higher level of utilisation of inpatient care among non-responders coincides with higher mortality and higher prevalence of serious chronic conditions in that group, thus if adjusted for need, the difference in utilisation could be smaller, or even reversed. Furthermore, the probabilities of general practitioner utilisation among responders and non-responders are comparable, despite the higher mortality rate and prevalence of important chronic diseases among non-responders, thus a contribution to overestimation of inequity in favour of the rich or educated is not likely, and a contribution to underestimation is possible. Being excluded from this study due to missing variables in HUNT3 was also correlated with low income and education, however no conclusions on the impact on estimates could be drawn due to missing data on health and utilisation.

The large variation in response levels between the young and the old represent potential bias in the intergenerational differences in inequity we found. If non-response is associated with underestimation of inequity, our estimates could exaggerate the gap in inequity between the young and the old for specialist services, given that the highest levels of inequity were found in the age group with the highest response rate.

Another limitation in the study is the self-reported nature of the health and utilisation data. The self-reported health data could lead to underestimation of pro-educated inequity if persons with high education systematically rate their health more negatively than persons with low education, which has been shown in one study [20]. This effect would be partly remedied by the various morbidity indicators included in the analyses, as they add a more objective aspect to the needs-adjustment than the self-reported health variable. The consistency in reporting of general practitioner utilisation found in the nonparticipation study makes systematic socioeconomic differences in reporting of utilisation less probable.

This study does not address the inequities in utilisation and treatment quality that might exist beyond the first instance of utilisation in a 12-month period. However, van Doorslaer et al. [21] found that in many European countries much of the overall horizontal inequity in specialist utilisation originates from inequity in initial contact. The inequity described in this study is thus likely to be an important part of the overall inequity in the health care experience of the population.

## Conclusion

Our data suggest that the utilisation of important health services like general practitioner and inpatient services, which have low access threshold or are free of charge, is equitably distributed in Norway. However, even in a country with a national goal of offering every citizen equal access to health services, we found systematic pro-rich and pro-educated social inequalities in utilisation for both private medical specialist and public hospital outpatient services.

## Competing interest

None.

## Authors’ contributions

EV processed, analysed, and participated in interpreting the data, wrote the first draft and finalised the manuscript. SK and SW proposed the study design, oversaw interpretation of data and revised the paper.
